# A Novel Approach of Pig Weight Estimation Using High-Precision Segmentation and 2D Image Feature Extraction

**DOI:** 10.3390/ani15202975

**Published:** 2025-10-14

**Authors:** Yan Chen, Zhiye Li, Ling Yin, Yingjie Kuang

**Affiliations:** College of Mathematics Informatics, South China Agricultural University, Guangzhou 510642, China; cheny@scau.edu.cn (Y.C.); yin_ling@scau.edu.cn (L.Y.); kuangyj@scau.edu.cn (Y.K.)

**Keywords:** pig weight estimation, image segment, image feature extract, deep learning, regression network

## Abstract

**Simple Summary:**

Accurate pig body weight is essential for feeding, health checks and farm profitability, yet traditional weighing is slow, costly and stressful for animals. We developed a low-cost, contactless method that uses ordinary top-down photographs to automatically isolate a pig’s back, extract simple shape measurements, and predict body weight. The image pipeline reliably finds the dorsal region, and the best model produced an average error of about 3.9 kg, with the majority of predictions within 5 kg of the true weight. Because the method uses only a standard camera and lightweight computation, it can be deployed on farms for frequent, noninvasive monitoring. This enables better feeding decisions, earlier detection of growth or health problems, reduced animal handling and lower labor costs, supporting improved animal welfare and more efficient, sustainable swine production.

**Abstract:**

In modern livestock production, obtaining accurate body weight measurements for pigs is essential for feeding management and economic assessment, yet conventional weighing is laborious and can stress animals. To address these limitations, we developed a contactless image-based pipeline that first uses BiRefNet for high-precision background removal and YOLOv11-seg to extract the pig dorsal mask from top-view RGB images; from these masks we designed and extracted 17 representative phenotypic features (for example, dorsal area, convex hull area, major/minor axes, curvature metrics and Hu moments) and included camera height as a calibration input. We then compared eight machine-learning and deep-learning regressors to map features to body weight. The segmentation pipeline achieved mAP_50_–_95_ = 0.995 on the validation set, and the XGBoost regressor gave the best test performance (MAE = 3.9350 kg, RMSE = 5.2372 kg, R^2^ = 0.9814). These results indicate the method provides accurate, low-cost and computationally efficient weight prediction from simple RGB images, supporting frequent, noninvasive monitoring and practical deployment in smart-farming settings.

## 1. Introduction

In modern animal husbandry, the swine industry is a critical pillar of the global food supply and agricultural economy [1]. A pig’s body weight is a vital physiological metric linked to growth rate, feed conversion efficiency, nutritional requirements, and overall health [2]. Therefore, achieving accurate, unobtrusive, real-time monitoring of pig body weight has immense practical value. However, traditional methods for measuring pig weight primarily rely on contact-based mechanical or electronic scales. These approaches not only require considerable labor for herding and weighing, but also readily induce stress responses in the animals. With rapid advances in computer vision and artificial intelligence, image-based, contactless livestock weight estimation has emerged as a highly promising research direction. This approach substantially reduces disturbance and stress to the animals, allowing measurements in a more natural state, while providing data with far greater real-time continuity compared to conventional methods.

In current studies, three primary approaches—3D point clouds, depth maps, and 2D RGB images—are used to extract features from pigs, which are then combined with machine learning or deep learning models to estimate body weight.

3D point-cloud–based techniques capture detailed morphological features of pigs and provide richer body-size parameters; these methods are highly robust to complex surface geometry and partial occlusions and can achieve high-precision measurements in dynamic environments, thereby substantially improving weight-estimation accuracy [3]. He et al. [4] employed 3D imaging and a regression network for contactless pig weight measurement; their study introduced an image-enhancement preprocessing pipeline and a BotNet-based regression network to accurately predict pig body weight. Li et al. [5] conducted experiments using morphometric measurements from 50 pigs as predictor variables in regression models, and their results demonstrated the accuracy and reliability of the Kinect v2 sensor for capturing body dimensions and estimating weight. Nguyen et al. [6] used a handheld, portable RGB-D imaging system to generate point clouds for each pig and estimate weight, subsequently comparing the performance of machine learning and deep learning models. Kwon et al. [7] proposed a deep-learning–based approach to rapidly reconstruct mesh models from pig point-cloud data and extract various measurements for real-time weight estimation, and they developed DNN models for weight prediction. Selle et al. [8] presented a 3D data-modeling method for swine production; by constructing a statistical shape model they quantitatively and visually analyzed variations in body shape, morphology, and posture, and using linear regression with volume as the sole predictor they achieved accurate weight prediction. However, 3D point cloud devices are relatively expensive, and the acquisition and preprocessing of point cloud data are more complex, requiring operations such as filtering, registration, and denoising, and impose substantial computational demands, which limits their suitability for large-scale, low-cost field deployment.

A depth camera can capture scene depth information in a single exposure, thereby generating a depth map corresponding to the RGB image. Condotta et al. [9] utilized depth images to predict the weight of live animals, employing a linear equation for estimation. Cang et al. [10] proposed an intelligent method for pig weight estimation using deep learning; they designed a deep neural network that takes a top-view depth image of a pig’s back as input and outputs the estimated weight. Fernandes et al. [11] developed a system that uses a depth camera to acquire pig body measurements and automatically predict body weight. Na et al. [12] developed a Raspberry Pi–based pig weight prediction system, in which a Raspberry Pi module segments pigs from captured depth images and extracts features from the segmented images for weight prediction. However, depth cameras have limited ranging accuracy and effective measurement range; when the pig’s surface texture is uniform or highly reflective, depth data are prone to missing values or noise, necessitating interpolation and depth-map restoration, which increases algorithmic complexity.

Weight estimation methods based on conventional planar RGB images employ deep learning or traditional image processing techniques to extract contour area, shape features, and color–texture information from top-view or side-view images of pigs. These methods require minimal equipment—only a standard camera—offering low cost and ease of large-scale deployment. Kaewtapee et al. [13] captured dorsal images of pigs using a camera and developed a pig weight estimation model based on regression analysis and artificial neural networks (ANN). Banhazi et al. [14] developed a single-camera system to detect pig body width, length, and area, and estimated weight using these parameters. Thapar et al. [15] acquired top-view and side-view images of pigs to measure body dimensions; the technique was specifically applied to Ghoongroo pigs, successfully predicting their body weights. da et al. [16] presented a method for pig weight prediction using 2D images in conjunction with computer vision and machine learning algorithms. They developed a multivariate linear regression model capable of automatically extracting morphometric data from images to predict pig body weight. Wan et al. [17] employed a monocular vision approach based on an improved EfficientVit-C model, integrating image segmentation, depth estimation, and an adaptive regression network to achieve rapid, accurate, and non-invasive pig weight estimation. Ji et al. [18] proposed a machine learning–based method for real-time pig weight estimation by extracting image features; the approach maintains high accuracy while reducing computational demands through on-the-fly image segmentation for feature extraction. However, pure 2D images cannot directly capture depth information and are highly sensitive to changes in lighting, background interference, and variations in pig posture, often requiring strict imaging conditions and calibration procedures; otherwise, feature extraction may become unstable, thereby affecting the accuracy of weight estimation.

The most prominent characteristics of traditional machine learning methods are their simplicity, broad applicability, and ease of use. With the advancement of mathematical regression techniques, feature parameters have evolved from one-dimensional representations to multidimensional forms. Integrating machine learning methods into the regression prediction process can significantly enhance prediction accuracy. Compared with traditional machine learning, deep learning algorithms can accurately and efficiently extract high-value features from high-dimensional and complex data, potentially yielding more accurate and reliable results [19].

Building upon the aforementioned studies and considering the practical characteristics of agricultural engineering requirements, both 3D point cloud and depth image methods, although demonstrating excellent performance, suffer from high equipment costs, stringent constraints on data acquisition environments, and significant computational resource demands. These limitations hinder their applicability in capturing pigs in their most natural states within complex environments and make lightweight deployment challenging. Therefore, this study adopts standard planar RGB images for predicting pig body weight. Using RGB images eliminates the need for expensive acquisition devices, simplifies data processing, ensures high data availability, and results in a low proportion of noisy data, thereby facilitating practical application in agricultural engineering. This study aims to address the following issues: In previous research utilizing planar RGB images, the prevailing approach has been a “one-step” strategy, in which images are directly subjected to recognition or segmentation without additional preprocessing. Moreover, the employed methods are relatively outdated, resulting in limited predictive accuracy [20,21]. In contrast, certain methods that achieve higher accuracy often do so by constraining the background environment and the posture of pigs [22], which reduces robustness and transferability. To overcome these limitations, this study proposes a “two-step” image processing pipeline: first, the background is removed to eliminate irrelevant interference, and subsequently, state-of-the-art instance segmentation models are applied to the target. In addition, camera height is incorporated as an independent feature, compensating for the lack of depth information in planar RGB images. Furthermore, a novel feature parameter, “body curvature,” is introduced to characterize pig posture; its interaction with other features enhances both accuracy and robustness. Finally, eight mainstream models, including traditional machine learning and deep learning approaches, are systematically compared, and the optimal solution for this study is identified.

The contributions of this work are as follows:

- Utilizing the publicly available dataset provided by [18], the study enables easy reproducibility and comparison, breaking the limitation of widespread reliance on proprietary datasets in the field of automatic livestock weight estimation.
- Innovatively integrating the latest high-resolution segmentation network (BiRefNet) [23] with the instance segmentation model (YOLOv11-seg), this approach enables precise extraction of the pig’s dorsal region under free-moving conditions in complex scenes without confining the animal to a fixed area.
- Based on previous studies and our analytical discussions, a total of 17 relevant features were identified and validated through ablation experiments. This not only enriches the feature set but also provides valuable references for future research in this field.
- A comparative analysis of eight mainstream models, encompassing both traditional machine learning and deep learning methods, ultimately confirms the superiority of ensemble tree models for this task.

This paper is organized as follows. Section 2 describes the dataset, presents the proposed method, analyzes the extracted features and provides a discussion of the model. Section 3 provides the experimental conclusions. Section 4 discusses this conclusion and compares the results of other papers. Section 5 concludes the paper.

## 2. Materials and Methods

### 2.1. Introduction to Dataset

#### 2.1.1. Dataset Source

**Figure 1:**
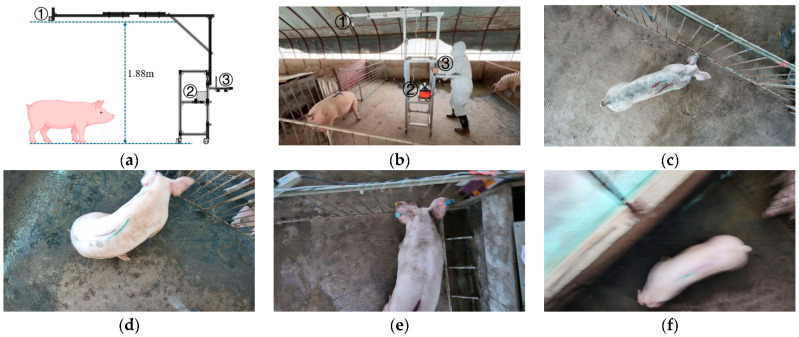
Dataset illustration. (**a**,**b**) depict the data collection process, Among them, ① is the camera, ② is the power, and ③ is the terminal [18], (**c**) shows the dataset, (**d**) demonstrates an incomplete dorsal image region caused by posture distortion, (**e**) shows an incomplete capture of a pig due to the camera shooting angle, and (**f**) illustrates image blurring caused by rapid movement of the pig.

The dataset used in this study is sourced from the publicly released data in the work of [24], which contains 12,972 top-view images of pigs’ dorsal regions, collected in the activity areas of a pig farm. The data collection procedure is as follows: first, pigs were driven out of the group pen and guided into a crate scale for weight measurement and recording. Next, the pigs were moved into an empty enclosure containing the image acquisition system, and the collection cart was adjusted to a suitable position to ensure that the camera was vertically aligned with the pigs’ dorsal region before starting data acquisition. During the collection process, the pigs were tracked while moving freely, and images were continuously captured until the target quantity was reached. Finally, the pigs were returned to the group pen (see Figure 1a,b). This collection method does not interfere with the pigs’ natural activities, thereby reducing stress and capturing them in their most representative natural state. The cameras were mounted at heights of 1.78 m and 1.88 m, with the imaging angle kept perpendicular to the pigs’ dorsal surface to minimize perspective distortion, and the images were standardized to a resolution of 960 × 540 pixels and stored in PNG format. The weight data of the pigs ranged from 33.1 kg to 192 kg, preventing overfitting caused by focusing on a narrow weight range; for each recorded weight, there were at least six images, with an average of over 100 images, providing the model with substantial sample diversity.

The dataset encompasses the following behavioral states of pigs:

- Upright: The pig stands erect, supporting its body with all four limbs, maintaining an upright posture with the head prominently extended.
- Feeding: The pig is engaged in feeding, typically observed foraging or eating from a trough.
- Walking: The pig moves within the activity area, with its limbs alternating in a regular, rhythmic manner.
- Drinking: The pig is drinking, characterized by lowering its head toward the water source.
- Abnormal standing: The pig exhibits an abnormal standing posture, with a twisted body or irregular stance.
- Head-lowered standing: The pig stands with its body upright but its head lowered.
- Partially occluded multiple pigs: The image contains multiple pigs, but parts of their bodies are obscured or incomplete due to the viewing angle or occlusion by other pigs or objects.
- Fully visible multiple pigs: The image contains multiple pigs that are fully visible without any occlusion or distortion.

These states reflect the most common real-life scenarios of pigs and do not induce stress.

Since no human intervention was applied to the pigs’ behavior during data collection, the images capture a wide variety of their natural postures. In addition, the spacious collection pen closely simulated the pigs’ daily living environment, allowing them to move freely. Consequently, the collected images accurately represent the pigs’ natural morphology and behavior, consistent with their everyday habits. The dataset is large in scale with a wide sample distribution, making it highly suitable for the present weight estimation task (see Figure 1c).

#### 2.1.2. Data Preprocessing

We backed up the dataset to local storage and first removed the original mask images provided, as we required masks containing only the pigs’ dorsal regions, which we intended to generate automatically rather than annotate manually. All images were organized into a single directory and annotated with basic information, such as weight and camera height. Subsequently, a small number of images that did not meet experimental requirements were manually removed. The criteria for image exclusion were as follows: (1) excessive body distortion of the pig resulting in an incomplete back region in the image, (2) shooting angles that captured only a partial view of the pig, and (3) motion blur caused by rapid movement (see Figure 1d–f). A total of 496 images were excluded based on these criteria. All excluded images were those that, upon discussion, were determined to lack a complete and reliable representation of the pig’s back region.

#### 2.1.3. Dataset Partitioning

After image preprocessing, a total of 12,476 images met the requirements, providing sufficient data for the experiment. Among these, 1500 images were randomly selected for learning the pigs’ dorsal region masks, with 1200 used as the training set and 300 as the validation set. The remaining 10,976 images were partitioned using individual pigs as the minimum unit of division, ensuring that images of the same pig did not simultaneously appear in the training, validation, and test sets. At the same time, the partitioning process maintained a uniform distribution of pig weights across the three subsets, with the number of images satisfying a 6:2:2 ratio. The final split was as follows: the training set contained 74 pigs with 6544 images, covering a weight range of 33.1 kg to 192.48 kg; the validation set contained 23 pigs with 2210 images, covering a weight range of 39.5 kg to 180.47 kg; and the test set contained 23 pigs with 2222 images, covering a weight range of 33.64 kg to 192.39 kg. Since a small number of pigs in the dataset had only a limited number of images, when randomly selecting 1500 images for learning pig back-region masks, all images of four pigs were included. Consequently, the total number of pigs in the training, validation, and test sets was reduced by four.

### 2.2. Pig Weight Prediction Based on High-Precision Segmentation and 2D Image Features

**Figure 2:**
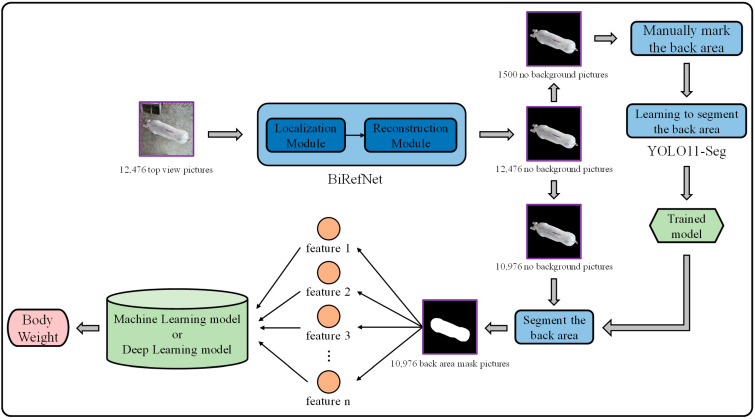
Technical Workflow Diagram.

The main methodological workflow of this study is illustrated in Figure 2. First, BiRefNet [23] was used to remove the background regions and obtain the foreground images. Next, a subset of images was manually annotated for the dorsal region, and YOLOv11-seg was used to learn these regions. The resulting model was then applied to extract dorsal region masks for the remaining images. Features were subsequently extracted from these dorsal region mask images, and finally, these features were used to predict weight by comparing the results of various machine learning and deep learning models.

#### 2.2.1. Remove Background

To accurately extract the pigs’ foreground regions, this study first employed the Bilateral Reference for High Resolution Dichotomous Image Segmentation (BiRefNet) framework [23] to perform background removal.

This method innovatively introduces a Bilateral Reference mechanism, in which the Localization Module (LM) and Reconstruction Module (RM) work collaboratively, while incorporating Auxiliary Gradient Supervision to enhance attention to detailed regions, thereby significantly improving both fine-grained segmentation accuracy and overall segmentation quality. In the Localization Module, BiRefNet utilizes global semantic features to generate a coarse foreground heatmap, which serves to approximately locate the pig’s body region. In the Reconstruction Module, the network treats hierarchical patches of the image as the source reference and the corresponding gradient maps as the target reference, using the Bilateral Reference mechanism to fuse multi-scale information and reconstruct a high-quality segmentation map [23].

The purpose of background removal is to eliminate potential interference from background regions during subsequent image processing and feature extraction, thereby improving the accuracy of these tasks while also facilitating manual annotation of the pigs’ dorsal mask regions.

#### 2.2.2. Dorsal Mask Extraction

In top-down images of pigs, the head and tail exhibit significant variability, and these variations can greatly affect image processing and feature extraction. However, the head and tail have minimal impact on the pigs’ body weight [25]. Therefore, in this study, the heads and tails were consistently removed from the images, retaining only the body region.

The dorsal regions of 1500 randomly selected images were manually annotated using the LabelMe software 5.2.1 [26], generating dorsal mask information while removing the pigs’ heads and tails. After annotation, the YOLOv11-seg model was trained on the annotated data. Compared to the latest mainstream segmentation model YOLOv8-seg and Mask-R-CNN [27], YOLOv11-seg has fewer parameters and higher accuracy, employing improved backbone and neck structures to enhance feature extraction capability, thereby achieving more precise detection while maintaining a balance between accuracy and performance. YOLOv11-seg produces masks or contour lines outlining the pigs’ dorsal regions without heads and tails, which is particularly useful for tasks requiring accurate dorsal shape information. At the same time, YOLOv11-seg achieves a balanced trade-off among accuracy, inference speed, and computational resource requirements, making it well-suited to the needs of our task as well as potential deployment in agricultural engineering applications. By using BiRefNet to remove background regions, both the accuracy of manual annotation and the performance of dorsal mask learning with YOLOv11-seg were greatly improved.

Since multiple non-target pigs may appear at the edges of the images, these non-target pigs are not recognized as background and therefore are not removed by BiRefNet. It is therefore necessary to eliminate interference from non-target pigs during dorsal mask extraction. This issue is addressed by using the max_det parameter in YOLOv11-seg, which retains only the detection box with the highest confidence, thereby excluding interference.

#### 2.2.3. Image Binarization

The extracted dorsal mask of the pig was retained and set to white, while the remaining areas were set to black, thus obtaining a binarized image containing only the pig’s dorsal region.

#### 2.2.4. Feature Extraction

After the image processing stage, the resulting images are black-and-white, with the white regions representing the pig’s dorsal mask and all other areas set to black. Next, using the white regions as the target areas, the following features are extracted: dorsal area, convex hull area, major axis, minor axis, body curvature angle, perimeter, maximum curvature, and Hu Moments.

Dorsal Area: The dorsal area of a pig is considered to be strongly correlated with its body weight [19]. After image processing, the area of the white region can be directly calculated to obtain the dorsal area.

**Figure 3:**
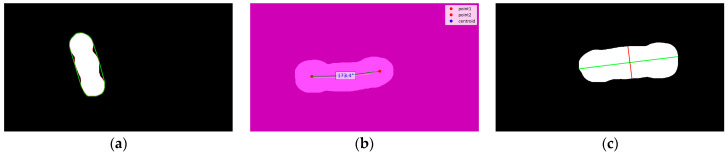
Illustration of Selected Features. (**a**) showcased the Convex Hull Area, the green line represents the smallest non-concave contour enclosing the mask region, the red line represents the external contour, (**b**) showcased the Body Curvature Angle, the two red dots are endpoints, the blue dot is the centroid of the skeleton, the yellow line represents the skeleton, and the green line represents the line connecting the two endpoints to the centroid, (**c**) showcased the Major and Minor Axes, the green line represents the Major Axes and the red line represents the Minor Axes.

Convex Hull Area (Figure 3a): The convex hull of the pig’s dorsal mask refers to the smallest non-concave contour enclosing the mask region. Calculating the convex hull area involves computing the area enclosed by this contour, which can be compared with the dorsal area. A smaller difference indicates less concavity in the dorsal region, suggesting smoother and potentially more robust body parts.

Curvature Angle (Figure 3b): The curvature angle of the pig’s back is not directly related to body weight, as pigs of any weight can freely bend their bodies. Moreover, the dataset was collected under the pigs’ common natural postures, without forcing them to maintain a straight body. However, the curvature angle affects the calculation of the convex hull area and the major axis. Since the convex hull measures the area of the non-concave region, larger curvature angles increase concavity, which in turn enlarges the convex hull area. At the same time, a larger curvature reduces the length of the major axis. Therefore, extracting the curvature angle makes the convex hull area and major axis features more accurate and effective.

Major and Minor Axes (Figure 3c): The geometric center of the mask region is determined, and the longest and shortest lines passing through this center within the mask are identified. These lines approximately represent the pig’s body length and abdominal width. The major and minor axes not only indicate the pig’s size but can also be used to estimate body fat by calculating their ratio, making them relevant features for body weight prediction.

Perimeter: The perimeter of the dorsal mask region can be obtained by directly calculating the length of the contour of the white area, as the perimeter of the pig’s dorsal contour is an important indicator of body size, which in turn is correlated with the pig’s body weight.

Maximum Curvature: A larger curvature value indicates a higher degree of bending in that region. By calculating the maximum curvature of the image contour, we can locate the point on the pig’s back contour where the curvature is greatest. We hypothesize that heavier pigs, due to their fuller and rounder bodies, may have a relatively smaller maximum curvature along their back contour. Therefore, this feature may capture body shape information that is related to body weight.

Hu Moments [28]: Hu moments are a set of seven invariants constructed from the geometric moments of an image region, used to describe the shape features of an image or contour. They are invariant to translation, scaling, rotation (and reflection), and are commonly used in pattern recognition and object matching. The calculation of Hu Moments is given in Formulas (1)–(11):

(1) $$M_{pq}=\sum_{x}\sum_{y}x^{p}y^{q}f\left(x,y\right)$$

(2) $$\bar{x}=\frac{M_{10}}{M_{00}},\bar{y}=\frac{M_{01}}{M_{00}}$$

(3) $$\mu_{pq}=\sum_{x}\sum_{y}{\left(x-\bar{x}\right)}^{p}\;{\left(y-\bar{y}\right)}^{q}\;f\left(x,y\right)$$

(4) $$\eta_{pq}=\frac{\mu_{pq}}{\mu_{00}^{\gamma }},\gamma =1+\frac{p+q}{2}$$

(5) $$\phi_{1}=\eta_{20}+\eta_{02}$$

(6) $$\phi_{2}={\left(\eta_{20}-\eta_{02}\right)}^{2}+4\;\eta_{11}^{2}$$

(7) $$\phi_{3}={\left(\eta_{30}-3\;\eta_{12}\right)}^{2}+{\left(3\;\eta_{21}-\eta_{03}\right)}^{2}$$

(8) $$\phi_{4}={\left(\eta_{30}+\eta_{12}\right)}^{2}+{\left(\eta_{21}+\eta_{03}\right)}^{2}$$

(9) $$\begin{array}{c} \phi_{5}=\left(\eta_{30}-3\;\eta_{12}\right)\left(\eta_{30}+\eta_{12}\right)\left[{\left(\eta_{30}+\eta_{12}\right)}^{2}-3\;{\left(\eta_{21}+\eta_{03}\right)}^{2}\right] \end{array}\;\;\;\;\;\;\;\;\;\;+\left(3\;\eta_{21}-\eta_{03}\right)\left(\eta_{21}+\eta_{03}\right)\left[3\;{\left(\eta_{30}+\eta_{12}\right)}^{2}-{\left(\eta_{21}+\eta_{03}\right)}^{2}\right]$$

(10) $$\phi_{6}=\left(\eta_{20}-\eta_{02}\right)\left[{\left(\eta_{30}+\eta_{12}\right)}^{2}-{\left(\eta_{21}+\eta_{03}\right)}^{2}\right]\;\;\;\;\;\;\;\;\;\;\;\;\;\;\;\;\;\;\;\;\;\;\;\;\;\;\;\;\;\;+4\;\eta_{11}\left(\eta_{30}+\eta_{12}\right)\left(\eta_{21}+\eta_{03}\right)$$

(11) $$\begin{array}{c} \phi_{7}=\left(3\;\eta_{21}-\eta_{03}\right)\left(\eta_{30}+\eta_{12}\right)\left[{\left(\eta_{30}+\eta_{12}\right)}^{2}-3\;{\left(\eta_{21}+\eta_{03}\right)}^{2}\right] \end{array}\;\;\;\;\;\;\;\;\;\;-\left(\eta_{30}-3\;\eta_{12}\right)\left(\eta_{21}+\eta_{03}\right)\left[3\;{\left(\eta_{30}+\eta_{12}\right)}^{2}-{\left(\eta_{21}+\eta_{03}\right)}^{2}\right]$$

Here, $f\left(x,y\right)$ denotes the pixel value of the image at $\left(x,y\right)$; p and q are non-negative integers representing the order of the moments; $M_{pq}$ denotes the raw moment, which is used to compute the centroid, with $M_{00}$ specifically representing the sum of image intensities; $\bar{x}$ and $\bar{y}$ denote the centroid coordinates of the image; $\mu_{pq}$ denotes the central moment, which eliminates the effect of translation; $\eta_{pq}$ denotes the normalized central moment, which eliminates the effect of scaling; and $\Phi_{1}$ to $\Phi_{7}$ represent the Hu invariant moments.

After extracting all features, we processed the features and finally summarized a total of 17 parameters to be used as input for model training:

- mask_area: Area of the dorsal region.
- Convex_Hull_Area: Area of the convex hull.
- difference: Difference between the dorsal region area and the convex hull area.
- dif/mask: Ratio of the difference between the dorsal region area and the convex hull area to the dorsal region area.
- body_curve: Bending angle of the back.
- perimeter: Perimeter of the dorsal region.
- outline_curve: Maximum curvature of the dorsal contour.
- longest: Long axis.
- shortest: Short axis.
- Hu_1-Hu_7: Hu Moments.
- height: Camera height.

#### 2.2.5. Model Selection

In this study, a total of eight different models, including both machine learning and deep learning approaches, were compared, with the goal of identifying the model that best suits the requirements of this research.

Multiple Linear Regression: Multiple linear regression is a classical supervised learning algorithm, which predicts outcomes by establishing a linear relationship between input features and the target variable. Its fundamental idea is to minimize the mean squared error between predicted and true values, thereby determining an optimal set of weight parameters.

Nonlinear Regression: Nonlinear regression models the complex relationship between input features and output variables by constructing a nonlinear functional form, representing this relationship as polynomials, exponential functions, logarithmic functions, or other nonlinear forms. Compared to linear regression, nonlinear regression can better fit highly complex mappings, but it has lower interpretability, and its parameter selection and optimization process are often sensitive to initial values. In this study, we employed Support Vector Regression (SVR) [29] with a radial basis function (RBF) kernel to model the nonlinear relationship between image features and body weight. The SVR model predicts the output y according to:

(12) $$f\left(x\right)=\sum_{i=1}^{n}\left(a_{i}-a_{i}^{*}\right)K\left(x_{i,}x\right)+b\;$$

(13) $$K\left(x_{i},x\right)=exp\left(-\gamma {\left|\left|x_{i}-x\right|\right|}^{2}\right)\;$$

$x_{i}$ are the support vectors, $a_{i}$, $a_{i}^{*}$ are Lagrange multipliers obtained by solving the SVR optimization problem, and b is the bias term.

Random Forest [30]: Random Forest is an ensemble learning method, which improves model generalization by constructing multiple decision trees and aggregating their predictions through voting (for classification tasks) or averaging (for regression tasks). This approach can effectively handle high-dimensional data, capture nonlinear feature interactions, and exhibit strong resistance to overfitting. Moreover, Random Forest can provide feature importance rankings, facilitating the understanding of each input feature’s contribution to the predictions.

XGBoost [31]: XGBoost is an enhanced algorithm based on Gradient Boosting Decision Trees (GBDT), which significantly improves training efficiency and generalization by incorporating techniques such as regularization, split-point optimization, and handling of missing values. XGBoost performs exceptionally well when handling large-scale, high-dimensional, and sparse data, and is particularly suitable for addressing regression and classification problems with pronounced nonlinear relationships.

LightGBM [32]: LightGBM is another efficient implementation based on Gradient Boosting Decision Trees (GBDT), which employs a histogram-based decision tree algorithm and a leaf-wise growth strategy, significantly enhancing training speed and memory efficiency. It supports direct input of categorical features, can handle large-scale datasets, and achieves predictive accuracy comparable to or even surpassing that of XGBoost.

SVM [33]: Support Vector Machine (SVM) is a widely used supervised learning algorithm for classification and regression tasks, which achieves effective sample separation by constructing an optimal hyperplane in a high-dimensional feature space. For regression tasks, SVM employs Support Vector Regression (SVR), using kernel functions (e.g., radial basis function) to map nonlinear relationships, making it suitable for small- to medium-sized datasets.

MLP: A Multi-Layer Perceptron (MLP) is a feedforward neural network that contains one or more hidden layers, each composed of several nonlinear activation units. By optimizing the weight parameters through the backpropagation algorithm, MLP can effectively capture complex nonlinear relationships among input features. Compared with traditional machine learning algorithms, MLP has stronger expressive power but is more sensitive to data volume and hyperparameter tuning.

TabNet [34]: TabNet is a deep learning model based on attention mechanisms, specifically designed for processing structured tabular data. Through sequential decision steps and sparse attention mechanisms, TabNet can automatically learn feature selection strategies during training while maintaining model interpretability. Compared with tree-based ensemble methods, TabNet demonstrates strong performance and end-to-end learning capability when handling high-dimensional, complex data.

## 3. Experiments and Results

In this experiment, we used a laptop equipped with a 2.10 GHz Intel(R) Core (TM) i7-14700HX processor, 32 GB DDR5 6000 MHz RAM, and an NVIDIA GeForce RTX 4070 Laptop GPU, running Windows 11 Home Edition 23H2. The experimental setup is cost-effective and readily accessible, making it suitable for practical deployment in small- to medium-sized farms and other agricultural engineering contexts.

**Figure 4:**
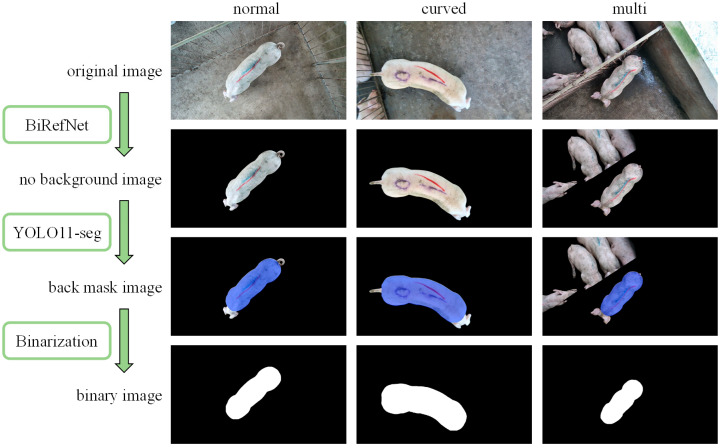
Data Processing Examples.

**Figure 5:**
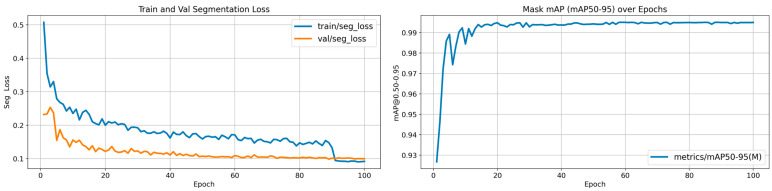
Training results of back segmentation.

First, the BiRefNet model pre-trained on the DIS dataset was applied directly to remove the background from the original images. Owing to the model’s excellent performance in complex and fine-grained scenarios and considering the satisfactory segmentation results obtained in this study, no additional training was necessary, as the model fully met the task requirements. Subsequently, 1500 background-removed foreground images were randomly selected for manual annotation of the back region and split into training and validation sets at an 8:2 ratio. The YOLOv11-seg framework was then employed for back region learning and segmentation. The training was conducted in a Python 3.8 and PyTorch 2.4.1 environment, using the official YOLOv11-seg pre-trained model, with an input resolution of 960 × 540, 100 training epochs, the optimizer set to “auto,” and all other parameters kept at default values. Upon completion of training, the back segmentation achieved an mAP50-95 of 0.995 in the validation set, which satisfied the requirements of this experiment. The results of data processing are illustrated in Figure 4, while the YOLOv11-seg training results for back segmentation are presented in Figure 5.

### 3.1. Evaluation Metrics

In this experiment, the evaluation metrics selected were the Mean Absolute Error (MAE), Mean Squared Error (MSE), Root Mean Squared Error (RMSE), and the coefficient of determination (R^2^).

MAE: Intuitively reflects the average deviation between predicted and actual values, using the same units and being easy to interpret. The calculation of MAE is given in Formula (14).

(14) $$\mathrm{MAE}=\frac{1}{n}\sum_{i=1}^{n}\left|y_{i}-\hat{y_{i}}\right|$$

MSE: Assigns greater penalty to larger errors, helping the model focus on reducing substantial deviations. The calculation of MSE is given in Formula (15).

(15) $$\mathrm{MSE}=\frac{1}{n}\sum_{i=1}^{n}{\left(y_{i}-\hat{y_{i}}\right)}^{2}$$

RMSE: Shares the same units as the original variables, making it easier to interpret compared to MSE. The calculation of RMSE is given in Formula (16).

(16) $$\mathrm{RMSE}=\sqrt{\frac{1}{n}\sum_{i=1}^{n}{\left(y_{i}-\hat{y_{i}}\right)}^{2}}$$

*R*^2^*:* Indicates the proportion of variance explained by the model, typically ranging from (−∞, 1], with values closer to 1 indicating better fit. The calculation of *R*^2^ is given in Formula (17).

(17) $$R^{2}=1-\frac{\sum_{i=1}^{n}{\left(y_{i}-\hat{y_{i}}\right)}^{2}}{\sum_{i=1}^{n}{\left(y_{i}-\bar{y}\right)}^{2}}$$

### 3.2. Experimental Results

To comprehensively evaluate the effectiveness of the feature set proposed in this study, we compared eight mainstream regression models. All models were trained, validated, and tested on the same dataset partitioned in a 6:2:2 ratio, with Grid Search employed to obtain optimal hyperparameters. The performance evaluation results of these models on the test set are shown in Table 1.

**Figure 6:**
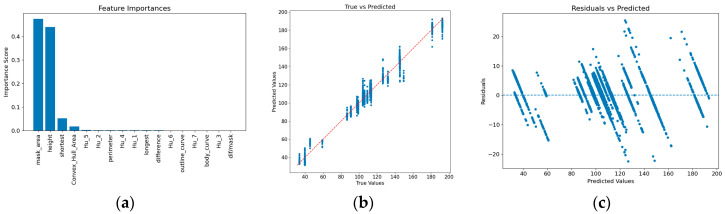
Visualization of the best-performing model. (**a**) Feature importance analysis, (**b**) Comparison between predicted and actual values, (**c**) Scatter plot of prediction residuals versus predicted values.

The experimental results clearly demonstrate that ensemble tree-based models, such as Random Forest, XGBoost and LightGBM, as well as deep learning models specifically designed for tabular data, like TabNet, significantly outperform traditional linear regression and support vector machine (SVM) models across all evaluation metrics. This indicates the existence of a complex nonlinear relationship between the phenotypic features extracted from pig images and their body weight. The four best-performing models achieved comparable results. Among them, the TabNet model yielded the lowest MAE (3.7711 kg), while the XGBoost model achieved the lowest MSE (27.4287 kg) and RMSE (5.2372 kg), as well as the highest R^2^ (0.9814). Based on these outcomes, XGBoost was selected as the optimal model in this study. To further investigate its performance, we conducted visual analyses of feature importance, prediction results, and residual distribution, as shown in Figure 6.

### 3.3. Preserve the Original Body Shape of the Pig

To verify the hypothesis proposed in Section 2.2.2 that “removing the head and tail regions and retaining only the main body of the pig’s back can improve prediction accuracy,” we conducted a comparative experiment. In this experiment, we re-extracted all 17 features using mask images that included the pig’s complete contour and trained and evaluated them with the same 8 models. The experimental results on the test set are presented in Table 2.

The results indicate that when the head and tail regions are retained, all models exhibit a consistent decrease in performance metrics. Taking the optimal XGBoost model as an example, its R^2^ decreased from 0.9814 to 0.9757. We attribute this to the fact that the posture of the pig’s head, ears, and tail varies greatly and has weak correlation with body weight; their inclusion introduces substantial noise into feature extraction. For instance, features such as maximum contour curvature and convex hull area are heavily affected by head rotation or tail movement, thereby weakening their correlation with the pig’s core body shape. This comparative experiment demonstrates that the strategy of segmenting only the back region, as proposed in this study, is both correct and necessary, effectively eliminating interference from irrelevant variables and improving the accuracy and robustness of weight estimation.

### 3.4. Removing Low-Importance Features

To investigate the contribution of each feature and validate the completeness of the selected 17 features, we designed further ablation experiments based on the feature importance ranking shown in Figure 6a. First, we removed the 8 least important features and retained only the top 9 features for model training, with the results on the test set presented in Table 3.

The experimental results demonstrate that, after removing certain low-importance features, the performance of most models declined to slightly degrees. This trend supports our hypothesis that, although some features have relatively low importance scores, they play a crucial fine-tuning role in the final prediction by providing subtle information that highly important features fail to capture. Notably, there is an exception: when the eight least important features were removed, the performance of the Nonlinear Regression model (SVR) improved. We argue that, first, SVR—particularly with an RBF kernel—depends critically on Euclidean distances in feature space. Irrelevant or noisy features dilute pairwise distances and thus degrade the discriminative structure of the kernel matrix; removing such features can therefore restore the kernel’s sensitivity to meaningful signal and improve generalization. Second, SVR is vulnerable to the curse of dimensionality in low-sample regimes: eliminating noise dimensions reduces variance and can substantially improve out-of-sample performance. Third, features with measurement error or extreme values can adversely affect the effective kernel scale (gamma); after removing these features, previously chosen hyperparameters may become more appropriate for the reduced-dimensional space, yielding better performance.

Subsequently, we conducted a more extreme test by retaining only the four most important features. This evaluation was carried out on the four best-performing models, as well as the model that showed improved performance after the removal of low-importance features in the previous experiment. The results on the test set are presented in Table 4.

The experimental results indicate that, whether for the models that originally achieved the best performance or for those that improved after the removal of low-importance features, retaining only the four most important features led to a decline in performance. This finding further validates our hypothesis that low-importance features play a critical fine-tuning role in prediction by providing subtle information that highly important features fail to capture. When only the high-importance features are preserved, the loss of such details results in reduced predictive performance.

### 3.5. Feature Correlation Analysis

**Figure 7:**
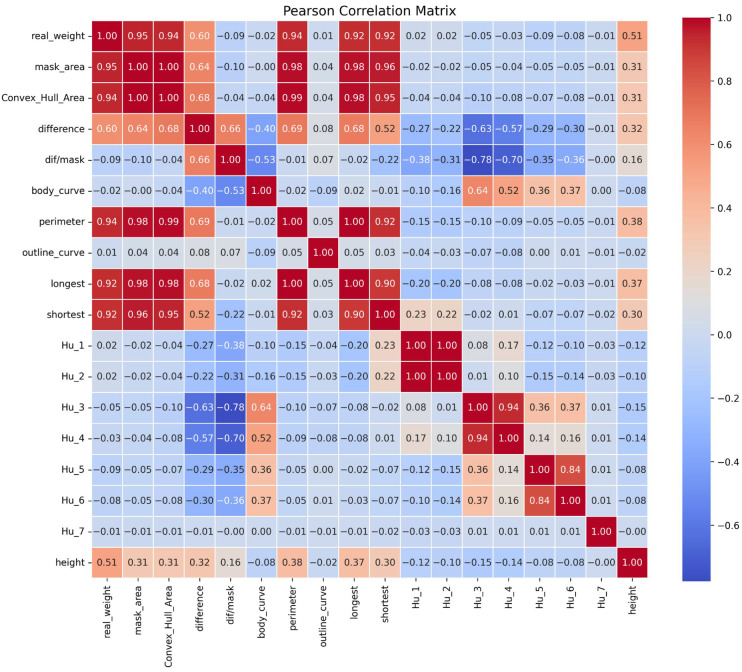
Heatmap displaying the Pearson correlation coefficients between each extracted feature and the pig’s body weight.

To gain a deeper understanding of the relationships between each feature and body weight, as well as the interactions among features, we calculated the Pearson correlation coefficients between all 17 input features and the real_weight, with the results presented in Figure 7.

Features that directly reflect pig size, such as mask_area (0.95), Convex_Hull_Area (0.94), perimeter (0.94), longest (0.92), and shortest (0.92), exhibit very strong positive correlations with actual body weight, which aligns with intuitive expectations and serves as the cornerstone for weight prediction. Additionally, height (−0.51) shows a moderate negative correlation, further confirming the necessity of including camera height as a key calibration parameter. Features such as body_curve and dif/mask have weak direct linear correlations with actual body weight, but they show significant associations with other features (e.g., the correlation between body_curve and difference is −0.53). This validates the design rationale discussed in Section 2.2.4: these “correction features” are intended to quantify deviations in core size features caused by postural variations such as body curvature, thereby indirectly improving model accuracy. The outline_curve and Hu Moment series (Hu_1 to Hu_7) generally show low linear correlations with body weight. However, considering the feature importance analysis in Figure 6a, these features—particularly outline_curve—contribute substantially to the final model. This indicates that these features may capture nonlinear shape information related to body weight, which cannot be measured by linear correlation analysis but is crucial for tree-based models such as XGBoost.

In summary, the feature correlation analysis further validates the rationality of the feature engineering in this study: the selected 17 features form a multidimensional and information-complementary feature space that encompasses direct predictors, indirect correction features, and nonlinear supplementary features, laying a solid foundation for subsequent high-precision modeling.

## 4. Discussion

For the XGBoost model, which achieved the best performance in the experiments, we applied it to predict the outcomes on the designated test set. Among the 2222 test samples, the body weights ranged from 33.64 kg to 192.39 kg, covering a broad spectrum and avoiding a focus solely on any specific weight range.

For these 2222 samples, the average difference between the true and predicted weights was 3.93 kg, meeting the requirements for agricultural engineering applications. Specifically, 416 samples had a predicted weight within 1 kg of the true weight, 1546 samples within 5 kg, and 2115 samples within 10 kg. Meanwhile, the average ratio of the difference to the true weight across all samples was 0.044, with 443 samples showing a predicted weight within 1% of the true weight and 1627 samples within 5%.

From Figure 6b, which shows the prediction results of the best-performing model, it can be seen that the predicted values exhibit a strong linear relationship with the true values, indicating a high degree of model fit. From the residual plot in Figure 6c, it is observed that the distribution of errors is not entirely random. In the regions where the actual weight is around 60 kg and above 170 kg, the residuals exhibit concentrated overestimation and underestimation, indicating that the model shows certain biases in weight estimation for pigs within these specific ranges. This phenomenon may suggest that the relationship between body shape features and weight varies subtly across different growth stages. This provides a direction for future work, such as developing stage-specific weight estimation models to further improve overall accuracy.

As shown in the feature importance plot in Figure 6a, the area of the back region accounts for an importance score of 0.474 in weight estimation. Therefore, the accuracy of calculating the back area can directly affect the precision of weight prediction. The method proposed in this study, which combines a high-resolution segmentation network (BiRefNet) with an instance segmentation model (YOLOv11-seg), achieves an mAP50−95 accuracy of 0.995 for the back region on the validation set, further enhancing the accuracy of weight prediction. It is also observed that camera height, short axis length, and convex hull area significantly affect weight. Due to perspective scaling, the camera height directly influences the captured area of the pig’s back, while the length of the short axis can indicate the pig’s fatness, with heavier pigs generally being fatter. The convex hull area additionally reflects the roundness of the pig’s body contour.

Considering the four parameters that have the greatest impact on weight, their relationships are not simply linear. For example, the relationship between camera height and back area requires complex mathematical functions to fit. Therefore, ensemble tree-based models (Random Forest, XGBoost, LightGBM) and the neural network-based TabNet model outperform traditional linear models overall. This provides a new direction for future research, suggesting that the adoption of such models may yield favorable results in estimating the body weight of poultry animals.

The performance of our method compared to other approaches using planar RGB images for pig weight estimation is shown in Table 5. First, compared with the article from which our dataset originates [18], under the same dataset conditions, our method achieves better MAE, MSE, RMSE, and R^2^. Specifically, MAE, MSE, and RMSE decreased by 10.31%, 22.60%, and 11.89%, respectively, while R^2^ increased by 1.70%, demonstrating the effectiveness of our BiRefNet + YOLOv11-seg image processing approach. Among the other compared models, three achieved higher R^2^ values than our method. In the study [22], the proposed method requires pigs to pass through a narrow corridor for image collection. Firstly, driving pigs through this corridor may induce stress responses. Additionally, the method discards images where the pigs’ bodies are slightly bent, keeping only fully straightened postures, which greatly increases the workload. In contrast, our approach does not require driving the pigs; data are collected while the pigs are in their most natural state, without imposing specific postures, resulting in higher robustness. In the study [35], the estimation is based on the weekly average weight per pen rather than individual pigs. Since pig weight trends follow a certain regular pattern, averaging the predicted weights per pen yields excellent results. In the study [36], a series of methods produced good results; however, the dataset was very small, with only 39 pigs concentrated between 104 kg and 138 kg, resulting in low model robustness and potential overfitting risks. In contrast, our study involves 124 pigs, with weights ranging from 33.1 kg to 192 kg, covering a wide range. This ensures high model robustness and strong resistance to overfitting.

Table 6 compares the performance of our planar RGB image-based pig weight estimation method with depth-image-based approaches. From the comparison, although our method does not reach the top performance reported, it consistently exceeds the average level. Additionally, our approach benefits from easy data collection, low cost, and high reproducibility, making it more practical for agricultural engineering applications.

In Table 7, we compare the performance of our planar RGB image-based pig weight estimation method with 3 D point cloud-based approaches. Considering the differences in datasets, our method achieves performance nearly comparable to the best results in the table. Although 3D point cloud methods can capture additional features and parameters unavailable in planar RGB images, their data acquisition and preprocessing are relatively complex, requiring filtering, registration, and denoising operations, as well as stringent environmental conditions. This makes the collected samples susceptible to noise, potentially leading to biased data and suboptimal outcomes. In contrast, our method is more robust, resistant to interference, and yields more stable results.

This study also has certain limitations. The first limitation is related to the data. The pig images used in this study all come from a single breed; however, different breeds exhibit variations in body fat percentage, body shape, and other characteristics [51]. Using the same model to predict the weight of pigs from other breeds may result in poor performance and limited generalizability. The second limitation concerns posture. The system cannot automatically identify “dirty data” such as extremely twisted pigs or incomplete back regions, which were manually removed in this study. Failure to exclude such data could lead to poor prediction results. Next, environmental conditions pose a limitation. The pigs in the dataset were generally clean, resulting in high recognition accuracy. If pigs have mud on their backs or if lighting conditions vary drastically, the accuracy of segmentation and feature extraction could be adversely affected. Finally, regarding camera height, variations in this parameter in practical farming scenarios may pose challenges, potentially leading to a sudden drop in recognition accuracy.

Based on this, we have the following plans for future work. First, we plan to expand the dataset by collecting data from pigs of different breeds to enhance the generalizability of our study. Additionally, at the beginning of the processing pipeline, a lightweight classification network could first identify the breed, and then switch to the corresponding model, thereby eliminating manual intervention in subsequent steps. Second, we plan to add an automatic “dirty data” removal step after the BiRefNet process. For example, images with pig detection confidence below a certain threshold could be removed (addressing extreme distortions or blurred images), and images where pigs touch the frame could also be removed (addressing potential incomplete pig regions). Then, since a high-accuracy back region mask extraction model has already been trained, transfer learning using only a small amount of additional pig data from other environments would suffice to adapt the model. Also, we plan to deploy the model on edge computing devices for on-site farm testing to verify its real-time performance and stability. Finally, regarding the issue of camera height, we propose the following considerations. If there is a global change in camera height, the model can be updated by retraining with modified feature values to obtain new model weights. Furthermore, when sufficient data are available, camera height can be treated as a continuous rather than a discrete parameter in the model. This approach allows the model to effectively accommodate any variations in camera height, thereby improving its adaptability and robustness.

## 5. Conclusions

In this study, we propose a simple computer vision–based method for estimating pig body weight, allowing real-time weight measurement from top-view images of pigs in their normal state on farms, requiring only the recording of the camera height. First, we use BiRefNet to remove the background from the top-view pig images and then apply YOLOv11-seg to segment the pig’s back mask. From these mask images, we extract features including dorsal area, convex hull area, body curvature, contour perimeter, contour curvature, major axis, minor axis, and Hu Moments. Using these features for prediction, the XGBoost model achieves the best performance, with MAE, MSE, RMSE, and R^2^ values of 3.9350, 27.4287, 5.2372, and 0.9814, respectively. Compared with other methods on the same dataset, MAE, MSE, and RMSE were reduced by 10.31%, 22.60%, and 11.89%, respectively, while R^2^ increased by 1.70%. Because our study relies solely on planar RGB images and employs only two simple networks for image processing alongside machine learning for weight prediction, the model architecture is compact, and both training and inference are computationally efficient. In summary, our method demonstrates good adaptability across pigs of different weights and can be easily and cost-effectively applied in practical agricultural settings.

## Figures and Tables

**Table 1:** Comparison of Performance Across Models on the test set.

Model	MAE	MSE	RMSE	R^2^
Multiple Linear regression	6.1705	82.8861	9.1042	0.9502
Nonlinear regression	4.1320	35.4693	5.9556	0.9759
Random Forest	3.9248	28.4137	5.3305	0.9807
XGBoost	3.9350	27.4287	5.2372	0.9814
LightGBM	3.9141	27.5670	5.2504	0.9813
SVM	4.9808	56.1862	7.4957	0.9618
MLP	5.0338	45.1849	6.7220	0.9693
TabNet	3.7711	28.0333	5.2946	0.9809

**Table 2:** Comparison of model performance when the head and tail regions are retained on the test set.

Model	MAE	MSE	RMSE	R^2^
Multiple Linear regression	6.5967	82.9126	9.1056	0.9496 ↓
Nonlinear regression	4.2722	37.3367	6.1104	0.9747 ↓
Random Forest	4.3826	34.2300	5.8506	0.9767 ↓
XGBoost	4.5927	35.7863	5.9822	0.9757 ↓
LightGBM	4.4569	34.3097	5.8574	0.9767 ↓
SVM	7.7819	120.0814	10.9582	0.9184 ↓
MLP	5.3156	53.2557	7.2977	0.9638 ↓
TabNet	5.0041	40.7700	6.3851	0.9723 ↓

“↓” represents a decrease in this data compared to the corresponding data in Table 1.

**Table 3:** Performance Comparison of Models After Removing the 8 Least Important Features on the test set.

Model	MAE	MSE	RMSE	R^2^
Multiple Linear regression	6.1574	83.4213	9.1335	0.9499 ↓
Nonlinear regression	4.1345	31.6546	5.6264	0.9785 ↑
Random Forest	3.9334	28.5761	5.3457	0.9806 ↓
XGBoost	3.9469	28.4734	5.0471	0.9806 ↓
LightGBM	3.9668	28.7848	5.3651	0.9804 ↓
SVM	6.0459	70.4073	8.3909	0.9521 ↓
MLP	6.7694	64.4057	8.0253	0.9562 ↓
TabNet	3.7760	28.2581	5.3158	0.9808 ↓

“↓” represents a decrease in this data compared to the corresponding data in Table 1, and “↑” represents an increase in this data compared to the corresponding data in Table 1.

**Table 4:** Results with only the four most important features retained on the test set.

Model	MAE	MSE	RMSE	R^2^
Random Forest	4.2404	31.5621	5.6180	0.9785 ↓
XGBoost	4.1471	30.9362	5.5620	0.9790 ↓
LightGBM	4.1784	31.3851	5.6022	0.9787 ↓
TabNet	4.1545	30.9646	5.5646	0.9790 ↓
Nonlinear regression	4.7644	45.2539	6.7271	0.9647 ↓

“↓” represents a decrease in this data compared to the corresponding data in Table 1.

**Table 5:** Comparison with the performance of other methods using planar RGB images.

Source	Dataset	Model	MAE	MSE	RMSE	R^2^
Ours	124 pigs, 33.1 kg–192 kg, 12,972 pictures	XGBoost	3.9350	27.4287	5.2372	0.9814
[18]	124 pigs, 33.1 kg–192 kg, 12,972 pictures (Same as the dataset in this article)	Backpropagation Neural Network	4.421	35.439	5.944	0.965
[16]	52 pigs, 41 kg–129 kg	SVR	0.12	---	0.28	0.91
[20]	40 pigs, 23 kg–45 kg	TF model	---	---	---	0.975
[21]	513 pigs, 70 kg–120 kg, 580 pictures	MLP	3.15	---	3.82	0.79
[22]	61 pigs, 14 kg–123 kg, 1000 pictures	ANN	---	---	---	0.9925
[35]	18 pigs, 20 kg–105 kg	Linear regression	---	---	---	0.99
[36]	39 pigs, 104 kg–138 kg,1505 pictures	XGBoost	0.389	---	0.576	0.995
[37]	73 pigs, 88 kg–132 kg, 456 pictures	VQTIM+LLE	---	---	---	0.8198
[38]	117 pigs, 70 kg–160 kg, 9800 pictures	Improve Resnet	---	---	3.52	---

**Table 6:** Comparison of the performance using depth-image-based methods.

Source	Dataset	Model	MAE	MSE	RMSE	R^2^
Ours	124 pigs, 33.1 kg–192 kg, 12,972 pictures	XGBoost	3.9350	27.4287	5.2372	0.9814
[9]	234 pigs, 8 weeks–21 weeks, 772 depth images	Linear regression	---	---	---	0.9907
[10]	20 pigs, 159.27 kg–167.27 kg, 19,978 depth images	VGG network	0.644	---	---	---
[11]	580 pigs, 21 nursery pigs, average weight 31.5 kg, 559 finishing pigs, average weight 119.8 kg	Stepwise regression	3%	---	---	0.92
[12]	15 pigs	Bayesian ridge regression model	---	---	10.702	---
[25]	132 pigs, 3.9 kg–104 kg, 2684 depth images	AdaBoost	2.96	12.87	---	0.987
[39]	251 pigs, 16 kg–130 kg	Nonlinear regression	---	---	1.8	0.98
[40]	10 pigs, 30 kg–110 kg, 1460 samples	Linear regression	---	---	---	0.9931
[41]	78 pigs, 6 kg–46.6 kg	Nonlinear regression	0.48	---	---	0.9942
[42]	more than 400 pigs, 20 kg–133 kg, 4179 depth images	ConvNets	---	---	3.8	0.978
[43]	427 pigs, 16.5 kg–117 kg, 58,138 depth images	Xception	1.16	---	1.53	0.9973
[44]	70 kg–125 kg, 15,466 depth images	two-stream cross-attention vision Transformer	3.237	---	5.993	0.742
[45]	74 kg–154 kg, 13,594 depth images	MPWEADV	2.856	---	4.082	0.901

**Table 7:** Comparison of the performance using 3D point cloud methods.

Source	Dataset	Model	MAE	MSE	RMSE	R^2^
Ours	124 pigs, 33.1 kg–192 kg, 12,972 pictures	XGBoost	3.9350	27.4287	5.2372	0.9814
[4]	29 pigs, 128,062 3D images	BotNet	6.366	---	---	---
[5]	50 pigs	ridge regression	2.961	---	5.079	0.958
[6]	733 pigs, growing pig weight: 25 kg–70 kg, fattening pigs: over 70 kg	SVR	9.25	---	12.3	0.418
[7]	70 pigs, 168 kg–313 kg, 1022-point clouds	deep neural network	4.8847	---	8.6899	0.9532
[8]	582 pigs, 50 kg–140 kg, 4315 images	Linear regression	2.837	---	---	---
[46]	23 pigs, 200 kg–300 kg, 137 data	Linear regression	2.43%	---	---	---
[47]	58 pigs, 83 kg–132 kg, 479 data	Linear regression	2.664	---	---	0.921
[48]	198 pigs, 190 kg–300 kg, 10,000 data	CNN	12.45	---	12.91	---
[49]	258 pigs	MACNN	11.81	---	11.552	
[50]	249 pigs, 2 kg–120 kg, 1186-point clouds	PointNet	---	---	6.87	0.94

## Data Availability

Restrictions apply to the availability of these data. Data were obtained from “PIGRGB-Weight” and are available [https://github.com/maweihong/PIGRGB-Weight (accessed on 24 June 2025)] with the permission of the author.
